# Bodily Complexity: Integrated Multicellular Organizations for Contraction-Based Motility

**DOI:** 10.3389/fphys.2019.01268

**Published:** 2019-10-15

**Authors:** Argyris Arnellos, Fred Keijzer

**Affiliations:** ^1^IAS-Research Centre for Life, Mind & Society, Department of Logic and Philosophy of Science, University of the Basque Country, San Sebastián, Spain; ^2^Department of Product and Systems Design Engineering, Complex Systems and Service Design Lab, University of the Aegean, Syros, Greece; ^3^Department of Theoretical Philosophy, University of Groningen, Groningen, Netherlands

**Keywords:** muscle, tissue contraction, animal sensorimotor organization, physiology, development, epithelia, animal evolution, Cnidaria

## Abstract

Compared to other forms of multicellularity, the animal case is unique. Animals—barring some exceptions—consist of collections of cells that are connected and integrated to such an extent that these collectives act as unitary, large free-moving entities capable of sensing macroscopic properties and events. This animal configuration is so well-known that it is often taken as a natural one that ‘must’ have evolved, given environmental conditions that make large free-moving units ‘obviously’ adaptive. Here we question the seemingly evolutionary inevitableness of animals and introduce a *thesis of bodily complexity*: The multicellular organization characteristic for typical animals requires the integration of a multitude of intrinsic bodily features between its sensorimotor, physiological, and developmental aspects, and the related contraction-based tissue- and cellular-level events and processes. The evolutionary road toward this bodily complexity involves, we argue, various intermediate organizational steps that accompany and support the wider transition from cilia-based to contraction/muscle-based motility, and which remain insufficiently acknowledged. Here, we stress the crucial and specific role played by muscle-based and myoepithelial tissue contraction—acting as a physical platform for organizing both the multicellular transmission of mechanical forces and multicellular signaling—as key foundation of animal motility, sensing and maintenance, and development. We illustrate and discuss these bodily features in the context of the four basal animal phyla—Porifera, Ctenophores, Placozoans, and Cnidarians—that split off before the bilaterians, a supergroup that incorporates all complex animals.

## Introduction

Multicellular systems evolved from unicellular ancestors on at least 25 independent occasions, both in prokaryotes and eukaryotes (Bonner, 2000). Of these 25 occasions, six eukaryote lineages gave rise to complex multicellularity. Complex multicellularity is characterized by intercellular adhesion and communication, and by (tissue) differentiation beyond the one between somatic and reproductive cells that is common among simple multicellular systems (Knoll, 2011). Within a (current) total of 119 eukaryotic clades, complex multicellularity originated three times in ‘plant-style’ lineages—leading to green algae and plants, red algae, and brown algae—as well as twice within the branch leading to modern fungi—leading to basidiomycetes and ascomycetes. Finally, complex multicellularity arose once within the animal (metazoan) lineage (e.g., Bonner, 1988; Medina et al., 2003; Knoll, 2011). Thus, while the evolution of complex multicellularity itself is not a unique evolutionary event, the evolution of animal-style multicellularity did happen only in one lineage. Accordingly, and based on previous work (Arnellos and Moreno, 2016; Keijzer and Arnellos, 2017), our goal in this paper is to further discuss and argue on the special organizational characteristics and the related bodily complexity necessary for the evolution of animal multicellularity.

Typical animals exhibit a free-moving motile lifestyle that allows them to actively change position with respect to the substrate they are situated on (or in), to actively manipulate their environment, ingest large chunks of foodstuff and so on.^1^ A similar free-moving lifestyle is widespread among unicellulars – whether they are bacteria or protists like ciliates or amoebae—and it seems an obviously adaptive lifestyle under a wide variety of conditions. Letting aside very complex behavior that involves increasingly complex nervous systems, the fundamental difference in comparison to these unicellular free-moving organisms is that many animals have extended such a motile lifestyle to much larger organizations, expanding from a submillimeter size to species measured in tens of meters, a difference of five orders of magnitude. It is for this reason that, superficially, typical animals may just seem to consist of larger, more complex versions of free-moving unicellulars, suggesting a gradual and piecemeal transition, similar to the later evolution of eyes (e.g., Nilsson and Pelger, 1994). Also, like plants, animal life is such a major component of modern macroscopic life that its presence may look like an unavoidable outcome of evolutionary pressures leading to a multicellular equivalent of the unicellular free-moving lifestyle (Conway Morris, 2003).

However, given (a) the obvious adaptive advantages of a free-moving lifestyle at small as well as at larger scales, as currently seen in typical animals, and (b) the presence of only one evolutionary lineage that actually led to complex free-moving multicellulars, an interesting option comes forward: The rise of typical animals did not consist of a straightforward gradual transition from small-scale to large-scale motility. The initial transition to the typical animal lifestyle was a very difficult one, which involved major transformations in organization, life-style and development (Arnellos and Moreno, 2016).

In particular, the organizational requirements for animals involved new emergent forms of complex integration implemented in bodies. As a matter of fact, animal multicellularity is unique in the sense that it exhibits a level of complexity that brings about typical bodies: differentiated, potentially large and relatively stereotypical multicellular organizations that are capable of self-initiated reversible motility—either as a whole or partially—and sensing as a single unit. This goes far beyond the capacities for self-initiated motility that are present in other multicellular organizations, especially with respect to the increase in force and speed enabled by contractions. It has been previously argued that the key difference that ultimately enabled the rise of typical animals was the development of integrated multicellular organizations implemented in complex body plans (Arnellos and Moreno, 2016) with motility based on tissues capable of reversible contractions, most notably muscle (Keijzer et al., 2013). In this paper, we posit and explain why and how contraction-based motility is one of the most characteristic and fundamental properties of large motile bodies, and we also show and argue for its indispensably mutual role in the development and maintenance of such specifically integrated multicellular organizations.

The use of muscle-based contraction was an essential condition for typical modern animals and the famous Cambrian Explosion, starting 542 million year ago, would not have been possible without it. During the Cambrian Explosion, animals with “complex adaptive bodies” – so called CABs by Trestman (2013) and centralized brains—’typical animals’—became the dominant fauna (Fedonkin et al., 2007). Complex prey-predator relations sprung up, possibly driven by the evolution of eyes (Parker, 2003) and reinforcement learning (Ginsburg and Jablonka, 2010). From here on the prominent story of animal evolution took off. The central animal lineage in these subsequent events were the *bilaterians*. This is a very large evolutionary group that is often defined in terms of having bilateral symmetry, a head, a tail, a back and a belly. They have a nervous system, muscles and a gut and include most commonly known animals, such as the vertebrates, arthropods, mollusks and many other groups.

The bilaterians thus represent a stage where the animal bodily organization exhibits the key features—muscle-based contractility, neural control, a gut, complex development and physiology—that made them so successful as motile multicellular organizations. In this paper, and considering, as mentioned above, previous work on the particularity of the form of integration and of the pivotal role of contractility in multicellular organizations, we further investigate *what set the basis for such complexly organized (animal) bodies.* Thus, here we focus on the space of more basic forms of animal organizations from which the bilaterians evolved. There are four known basal phyla that split of before the bilaterians: Porifera (sponges), Ctenophora (comb jellies), Placozoa, and Cnidaria (jellyfish). These basal phyla constitute a major source of information concerning the various forms of basal animal organization. We will sketch a range of configurations for bodily complexity to illustrate the intrinsic difficulties involved in functionally integrating many different organizational factors and keeping them coordinated. The typical animal body and its multicellular reinvention of the free-moving ‘unicellular’ lifestyle is only one of these options.

More specifically, in this paper, we focus on the various conditions that must have been fulfilled for typical animal bodies to become a reality. For this reason, we formulate a *thesis of bodily complexity*: The multicellular organization characteristic for typical animals requires the integration of a multitude of intrinsic bodily features between its sensorimotor, physiological, and developmental aspects, and the related contraction-based tissue- and cellular-level events and processes. As we argue, at least at such levels of organizational complexity, many of these conditions are best seen as being constrained by the requirements imposed by the specific form of integration of the bodily organization itself, and much less, if not at all, by environmental features acting through selective pressures. Only by first fulfilling a wide array of such organizational requirements did the animal multicellular configuration eventually—and only in some lineages—become capable of regaining the free-moving lifestyle of many unicellulars in a highly successful way. The initial hurdle here was not to gain more complex behavioral capacities, but to reacquire behavioral capacities already present in organisms using cilia, but now based on (muscle) contraction.

The *thesis of bodily complexity* highlights the complex routes traversed through that space of organizational forms of animal multicellularity, and the various ways of integrating contraction-mediated sensorimotor, physiological, and developmental aspects into functional organisms. We have three objectives. *First*, we aim to clarify the various features that are central to bodily complexity. These features are often taken as background structures in selection-oriented accounts. Here, we stress their role as basic requirements for the kind of functionality that selection might work on. *Second*, we will develop and discuss these features by turning to the various ways in which they come together and are integrated in the four basal phyla that split off before the bilaterian supergroup: Porifera, Placozoa, Ctenophora, and Cnidaria. Animals of these groups show interesting mixes of the various features and help to illustrate why the animal organization that is often taken for granted as a self-evident form of good design results from a complex integration of bodily features in ways that are not self-evident at all. As a *third* and additional aim, we hope to show how this mixing and integrating of features paved the way to the bodily complexity that eventually lead to the Cambrian Explosion.

The paper is structured as follows: In section two, we introduce the several features that play crucial roles in the origins of animal multicellularity and the forms of complexity present in animal bodies. The guiding thread here will be the role played by contractile tissues and cells (of muscular nature, in general) and its impact on bodily complexity as this is realized based on the integration between physiological, developmental, and sensorimotor processes in a multicellular system. The next four sections treat in detail the four different ways in which these features have been integrated in the four different basal phyla that are still extant today: Porifera, Placozoa, Ctenophora, and Cnidaria, again with an idea to the role played by contractile cells and tissues and the related form of integration achieved between development, maintenance and behavior. We compare the four phyla to the typical animal organization based on their organizational forms. Finally, in the concluding section we draw our conclusions concerning the major questions addressed, and develop our claim that establishing bodily complexity itself was a major evolutionary hurdle that cannot be seen as a reaction to evolutionary pressures. We argue that the key to understand the emergence and evolution of animals is to be found also at the level of the respective *bodily complexity*, i.e., at the properties and the characteristics of the early multicellular organizations exhibiting contraction (muscle)-based motility and at the related organizational requirements for the development and physiological maintenance of their integrated bodies.

## Setting the Stage for Bodily Complexity: Contraction-Based Motility Requires Integrated Bodies and Vice-Versa

### Epithelial Contractility and the Animal Sensorimotor Organization (ASMO)

Animals are not simply scaled-up unicellular agents but exhibit new and complex multicellular organizations that could have taken many different forms (Arnellos et al., 2014). Typical animal organizations require the fulfillment of a broad range of preconditions in order to develop and function. The switch to contraction-based motility is a crucial and fundamental precondition for several reasons. First, this switch enabled a much more forceful and fast form of motility that allowed the rise of (typical) animals. Second, coordinating contractions imposes an evolutionary need for tissues that control these contractions, for example excitable epithelia (Mackie, 1970) and, most notably, nervous systems (Pantin, 1956; Keijzer et al., 2013). Third, coordinating contractile tissue by means of a basic nervous system arguably provides a route toward new multicellular forms of sensing and multicellular sensors that work as single units (Arnellos and Moreno, 2015; Keijzer, 2015).

This latter capacity for sensory feedback derived from self-initiated contraction-based activity across a body surface forms the kernel of what we call the *animal sensorimotor organization* or ASMO. Keijzer and Arnellos (2017) specify the following conditions: (1) a multicellular body, constituting an ‘inner space’ or domain, which is differentiated from the body’s ‘outer space’ or environment; (2) the presence of contractile epithelia; (3) complex, standardized body architectures; (4) sensitivity to tension and stress at the level of (intra) cellular processes; and (5) reversible, contraction-based changes in body-shape. Here, we will use the ASMO as the key condition for typical animals.

The ASMO is an evolutionary key transition because it ties (literally) large collections of cells into a single motile unit, turning it into a large-scale structure of tensile and compression resistant elements that can act as a motile multicellular agent of macroscopic size. This amounts to a soft-bodied precursor of a tensegrity structure of hard skeletal struts and opposing muscles and tendons (Turvey and Fonseca, 2014). Such an agent can access its environment by multicellular senses that are sensitive to patterns of stimulation across surfaces and guide its own motility on the basis of such information (Keijzer, 2015). This organization is so familiar to us that it becomes almost invisible and hard to appreciate as a separate achievement of life. Still, it amounts to a major and specific evolutionary transition.

From here on, many evolutionary innovations can be made, as we can witness in the multitude of forms of typical animals. The important message that we want to bring across here is that a ‘typical animal’ configuration depends for its existence on the initial fulfillment of a broad variety of bodily constraints integrated in a specific organizational form in order to function as a free-moving agent (Arnellos and Moreno, 2015). These include controlled (muscle-based) contractions that actually achieve reliable motion and enable macroscopic feeding guided by sensory devices. Larger and differentiated bodies require various internal transport systems and an ongoing control of physiological processes, as well as a developmental system that guides cell differentiation and produces these complexly integrated multicellular organizations. So, while typical animal bodies are a very successful evolutionary invention, their initial evolution seems to depend a lot on the initial evolution and reciprocal calibration of these bodily features. The major asset of typical animals—free multicellular motility—only became established when these bodies were already in place.

The ASMO notion introduced above provides a suitable point within the space of possible animal configurations, and in particular, it provides a set of minimal conditions that form the basis for the behavioral characteristics of typical animals—muscle-based motility, complex sensory devices, and a centralized nervous system. Beginning by the empirical^2^ background of ASMO (Keijzer and Arnellos, 2017)—namely epithelial cell and tissues—we will focus on the evolutionary route toward an ASMO and the various ways in which basal phyla show different forms in which the various ingredients were combined and integrated in functioning organisms.

Epithelia provide the central constructive material and developmental scaffolding for much of the animal body (Tyler, 2003). They are two-dimensional sheet-like tissues, made up from epithelial cells connected to one another by a variety of specialized molecules (Tyler, 2003; Magie and Martindale, 2008). The cytoskeletons of epithelial cells are linked by these connections and the sheet can impose (contract) and resist mechanical tensions in various ways. Epithelial cells have an outward-facing apical side with a primary cilium, and a basal side anchored in an underlying extra-cellular basal membrane, which is part of the underlying extracellular matrix (ECM). The latter is a continuum of collagen fibers and fibrils that integrates and reinforces the contractile epithelium, making it more resistant to tension and provides a stable setting for the cells (Timpl, 1996). As epithelia provide the basic animal building block, they provide a plausible starting point for tracking the changes leading to complex animal bodies. Epithelial tissues are central to multicellular integration and when widely interpreted may constitute a very primitive multicellular configuration on its own. As a matter of fact, even the most minimally complex epithelial unit can be considered a body that exhibits integration between sensorimotor coordination, physiology, and development (Arnellos and Moreno, 2016).

In this context of contractile epithelial cells and tissues, we will develop two leading ideas. First, while contractility is a basic feature of eukaryotic cells that is directly linked to the presence of a cytoskeleton and widely used, turning contraction into a multicellular motility device is not an easy transition. It involves traversing what Kauffman (1993) calls a rugged fitness landscape with many local optima. Second, when it occurred, the switch from motility by cilia to contraction (muscle)-based motion was a central driving factor for the evolution of the ASMO: Using cellular contractions for motility requires a large number of organizational features in order to function. Both ideas will be developed by a systematic discussion of the main organizational characteristics of the four known basal phyla. The first one deals with the ways in which contractility is present in these phyla and is involved in the related constitutive and interactive functions. The second idea targets the ways in which the relevant organizational aspects like development, physiology and sensorimotor coordination guided by the presence of a nervous system—together with their integration to various animal body plans—correlate with and are potentially driven and mediated by contraction (muscle)-based motility. We provide here a short and general introduction of the two ideas and of the related features, while in the next four sections we will specifically explain how these features are exhibited and implemented in the four basal phyla.

### Contraction-Based Motility

Active motility plays a key feature in animal interaction because it allows the repositioning of the organism and environmental features with respect to one another. While cilia are extremely important and handy features as a motility device they are limited to small-sized organisms of a few millimeters, barring some specially adapted organisms like Ctenophora (Tamm, 2014). For large-scale motility as exhibited by typical animals, a different mechanism is required that comes in the form of tissue contraction, most notably muscle. Tissue contractions provide a much larger source of force that can be scaled to large organisms in the centimeter to meter range. The use of tissue contraction for motility does also require a much more complex coordination mechanism compared to cilia and this need for coordination will be our main focus in the following sections. A central idea here is that contraction control was a major driver toward the evolution of sensorimotor coordination as specifically realized through nervous systems.

### Contractility and Modes of Feeding

An implication directly related with motility in animals is feeding. As heterotrophs, organisms within the animal lineage acquire their energy and constitutive substances from the intake of food particles. A motile body has some interesting ways to be fed. Sperling and Vinther (2010) differentiate three general modes of feeding that, barring some derived exceptions, cover the whole animal lineage. Contraction-based motility characterizes all modes as it mediates between the animal and its food source in the environment in all three feeding types (though in different forms and degrees as discussed below). Microphagy – the direct uptake of bacteria and dissolved organic carbon from the water column – is found in Porifera. Placozoa feed with a ventral sole where the area between the animal’s ventral side and the underlying substrate acts as an ‘external stomach’ where food is externally (pre)digested before it is absorbed by the ventral surface. All other phyla – Ctenophora, Cnidaria, and the Bilateria – exhibit macrophagy. They take in large food particles, which are then internally digested in a gut. Interestingly, the presence of a gut goes hand in hand with the presence of both muscle and a nervous system, which suggests a strong connection between these features.

Another central idea here is that contraction-based motility provides behavioral implications such as the modes of feeding discussed before, which in turn entail organizational requirements related with sensorimotor, physiological, and developmental aspects of the animal body.

### Contractility and Sensorimotor Coordination (Coordinating Sensors and Effectors)

In an epithelial body exhibiting reversible contraction-based changes in body-shape together with sensitivity of environmental stimuli and stress at the level of each individual contractile epithelial cell, sensorimotor coordination happens primarily through the epithelial structure itself. This is characteristic for animals like the Porifera and the Placozoa, whose bodies are in constant and total contact with the water, and whose modes of feeding require an external or a cell-dependent micro-digestion. In these cases, epithelia (either at tissue level or as individual cells) function as sensor and effector structures. As the multicellular body becomes more robust with respect to its shape, and capable to implement more complex movements—especially with respect to finding and catching food—there is a need for more efficient and elaborated sensorimotor coordination. In such cases (e.g., Cnidaria, Ctenophora) where preys are hunted, caught, directed into a mouth and then pushed down into a gut, specialized cells stimulated by small ‘organs’ such as statoliths or explosive cells such as cnidocytes, and myoepithelia and/or true muscle cells function as effector structures, while coordination between sensors and effectors is implemented through a nervous system—i.e., a cellular tissue that enables the plastic and selectively combinatorial coordination of distant contractions in a multicellular body. Again, it is noted that macrophagy—having a gut—is always empirically combined with muscle-based motility and the presence of a nervous system (with various degrees of centralization).

### Physiology

The coordination of sensors with contractile effectors enables movements with various degrees of freedom for the animal, which in turn provides a context where physiological housekeeping processes related to the material, energetic, and metabolic requirements of such movements become relevant. As mentioned before, a fundamental and characteristic feature of animal bodies is their epithelialization due to which their cells are held together, oriented and attached to an ECM. This creates a three-dimensional tissue that separate an inner (systemic) and an outer (environmental) space with various degrees of sealing in between these two domains and with intercellular means of molecular, electrical, and signal communication between the cells of the tissue. In these three-dimensional bodies some cells will be in contact with the environment while other cells will be secluded in the inner space. This implies certain novel requirements for the transport of oxygen and nutrients required by all cells. Considering the low solubility of oxygen in the water and that that cell size should be restricted to ∼1 mm when oxygen availability is limited by diffusion, there is a challenge for 3-dimensional multicellular bodies to maintain the appropriate oxygen supplies (Knoll, 2011). Sperling et al. (2015) suggest this can be achieved either by maintaining body shapes that minimize diffusion distance or by evolving oxygen-transport systems. All basal metazoa have implemented the first strategy.

### Contractility and Development

Body motility in combination with a certain physiology requires a division of labor that cannot be achieved but through different cell types.^3^ In the transition from microphagy to macrophagy, multifunctionality and its subsequent segregation to sister cell types seem to have played an important role (Arendt, 2008). In this context, apart from the primary division between germ and somatic cells, epithelial, muscle, sensory, and neuronal cell types as well as some other very specialized ones (such as the cnidocytes and the nematocysts in Cnidaria or the collocytes in Ctenophora) were evolved from a cell type that most likely resembled a choanoflagellate.

The diversification of cell types as well as their differential constitution and spatial arrangement in a three-dimensional body requires certain forms of developmental regulation. In the evolutionary scenario from a colonial multicellular choanoflagellate to a metazoan, where the cellular functionalities of the latter are more or less encoded within the choanoflagellate genome (King et al., 2008), there is no need for evolution of different cell types but for the constant appearance of cell types that in their unicellular phase were alternating in time as well as for their spatial differentiation and arrangement within the three-dimensional body. In simple multicellularity, gradients of available light, oxygen, and nutrients of the environment together with signals transduced from exterior cells may induce differentiation of interior cells along the gradient (Schlichting, 2003). In more complex multicellularity, where, in general, development begins with a fertilized egg (Newman, 2014), it is epithelia^4^ that —provide a reliable scaffold to guide diffusing morphogens that initiate developmental processes, which in turn combine various (contraction-mediated) morphogenetic movements with mechanisms of inter- and intra-cellular signaling that mobilize and drive morphogenesis (Newman, 2012).^5^ To this, a diverse developmental signaling and transcription factors repertoire that seems to be present in all early metazoa plays a great role (e.g., Srivastava et al., 2008, 2010). This repertoire seems to have preceded the genomic developmental toolkit of bilaterians (Degnan et al., 2009; Riesgo et al., 2014), let alone the fact that this toolkit is not only present in the early metazoan genome but it is also expressed during their development.^6^

### Contraction-Based Motility and Integrated Bodies

We argue that enabling contraction-based whole-body motility—which in turn makes large and fast-moving animals possible—is not a self-evident evolutionary development but intrinsically tied up with an array of interdependent and crucial conditions for it to function. Together these conditions constitute the requirements for an integrated multicellular organization that involves the need to coordinate these contractions and to use the environmental sensory feedback they create (ASMO), the need to build complex and standardized multicellular bodies by a set of complex developmental processes, the need to maintain some chemical interaction space with the environment (such as a gut) for the organism as a whole, and the need for a broad array of physiological and homeostatic maintenance processes.

All these features are organized in (sometimes) fundamentally different ways in the four early diverging metazoan bodies. We argue that the ways in which *bodily complexity* can be said to increase (or at least differs) over these four phyla supports the idea that organizing contraction (muscle)-based free motility requires a highly complex integrated multicellular organization. These four phyla show how contractile capacities are present in all cases, while their use and characteristics differ in important ways. Enabling contraction as the primary source of motility, switching away from the use of cilia, occurs only in one of these four phyla. The discussion on these various uses of contraction and the specific ways in which this use is deeply connected to feeding-style (and general behavior), development and physiology show that the evolutionary route toward the replacement of ciliary by contraction-based motility is not a self-evident evolutionary improvement at this stage of multicellular organization.

In the following four sections we will describe in detail the different bodily complexities as they are materialized in each one of the form of integration of these four phyla with a particular focus on the presence and use of contraction for organizing body plan and motility. In all four animal cases, such use is intrinsically related to a specific organizational context, which sets its own restrictions on evolutionary changes.

## Porifera

Multicellular suspension feeders like the *Porifera* (sponges) have a water canal system (a network of chambers containing flagellated cells—the choanocytes) whose beating pumps water through the body. The choanocytes are then filtering bacteria (even less than 1 μm in size), which are digested intracellularly, making this a form of microphagy.^7^ But even in this form of feeding contractility plays an important role as sponges are able to contract their canals (slow contractile events that propagate through cellular sponges) so as to arrest the feeding current by closing the intake system in order to prevent damage to the feeding chambers and/or to expel inedible material that has entered the filtration system (Leys, 2015). Such behavior is enabled by the bodily organization and its tissue- and cell-related components.

### Cell Types

In Porifera there are around 11–16 cell types (Simpson, 1984; Bell and Mooers, 1997; Carroll, 2001). Few of them are found in the two spatially differentiated epithelia (the pinacoderm and the choanoderm) with different physiological roles in filtering, maintenance, and structural integrity of the animal (Leys et al., 2009). The pinacoderm consists of pinacocytes that make up the external surface (exo-pinacoderm) and the water channels (endo-pinacoderm), and porocytes that are miniature sphincter cells.^8^ The choanoderm is the feeding epithelium composed by choanocytes that have a single flagellum surrounded by a ring of microvilli.^9^ The two epithelial layers surround the mesohyl, which is a collagenous non-epithelial layer that provides the gross morphology of the sponge. It is primarily composed of an ECM secreted by motile amoeboid cells, and of archeocytes that can differentiate into all other sponge cell-types. Among these cell types are the lophocytes that secrete collagen fibers supporting the mesohyl as they move through it, the spongocytes and the sclerocytes that polymerize into thick skeletal fibers and secrete the mineralized skeletal spicules in many sponges, respectively. There are also the oocytes and the spermatocytes, which are reproductive cells undergoing gametogenesis to form sperm and eggs.

### Sensorimotor Coordination

In *sponges*, epithelia function as sensory and contractile tissues, and they are the primary conduits of behavior coordination (Nickel et al., 2011; Leys and Hill, 2012). The osculum (via the short, non-motile, primary cilia that line its inside) seems to be the main sensory organ for sensing environmental stimuli that trigger responses by the sponge (Ludeman et al., 2014). The effectors are either the flagella of the choanocytes (in the case of glass sponges)^10^ or contractile epithelial cells (i.e., actinocytes)^11^ that reduce the water flow into the sponge by reducing the size of the canals and chambers, or ‘sphincters’ formed by other specialized pinacocytes arising from the inner epithelium enabling thus the narrowing of the canal, and consequently, the prevention of the uptaking of unwanted particles that might damage their microvilli-based filter.

Sponge larvae first swim and then settle on a solid surface before their metamorphosis into a juvenile sponge. Each cell of the larval epithelium possesses a single, motile cilium and its apical end facilitates larval dispersal through swimming or crawling. Phototactic swimming seems to be the combination of the rotation of the larval body around its AP axis (due to metachronal beat of the cilia) and the shading by pigment of a region of cilia (Leys and Degnan, 2001). In some cases, phototactic responses of sponge larvae are the result of independent responses (either photonegative or photopositive) of individual cells (operating both as sensors and effectors) in the ciliated posterior tuft of the larva (Maldonado et al., 2003; Collin et al., 2010).^12^

Adult sponges coordinate the contractions of their canals (or the arrest of their flagella in the case of glass sponges) in order to prevent the damaging of their filter system by the uptake of large particles. In the absence of a nervous system there must be some other type of spatio-temporal coordination of the adult sponges’ cellular activities in order to detect and respond to the changes in water quality. Sponges are basally epithelial animals. The sponge larva consists of polarized epithelial cells, and adult sponges exhibit spatially differentiated epithelia with different physiological roles in filtering, maintenance, and structural integrity of the animal (Leys et al., 2009; Leys and Hill, 2012). The inner epithelium (*the endopinacoderm*) has a sensory function. Specifically, the osculum is the part of the inner epithelium that (via the short, non-motile, ‘primary’ cilia that line its inside) seems to be the main sensory organ for sensing environmental stimuli that trigger responses by the sponge (Ludeman et al., 2014). The sensory signals received by the cilia propagate to the rest of the sponge body mainly through cells of the epithelium. Sponges lack nerve cells in the sense that they cannot transmit signals along several cells to communicate to other cells via a chemical synapse. However, sponge cells send chemical signals in neighbor cells but in very low speeds compared to the conventional neuron-based transmission.^13^ Only glass sponges (Hexactinellida) have electrical signals conducted through the syncytium that trigger the halt of beating of the flagella of choanocytes in order to stop the feeding current.^14^ This has an ‘all or none’ effect (Leys and Mackie, 1997). Sponges of all other groups have no electrical signals. In these cases, the effectors are contractile epithelial cells (actinocytes) that reduce the water flow into the sponge by reducing the size of the canals and chambers, or ‘sphincters’ formed by other specialized pinacocytes arising from the inner epithelium enabling thus the narrowing of the canal. All these cases of coordination between environmental stimuli and effector contraction are realized through juxtacrine signaling (namely, a type of cell signaling where a signal released by a cell triggers a response in a neighboring cell (see Leys, 2015 for details). In all, epithelia in sponges function as sensory and contractile tissue, and they are the primary conduits of behavior coordination.

### Physiology

Porifera are in constant contact with the water through their highly porous bodies, while the flagellar movements of cells facilitate the circulation of water through the internal canals. Practically, the whole animal is within its environment. Nutrients are transported by the water that flows through the system of canals, whereas the exchange of nutrients takes place directly at the cellular level (microphagy).

Signal propagation through juxtacrine signaling is made possible due to sponge epithelia being able to control the ionic milieu of their extracellular space. Particularly, the outer epithelium (the pinacocytes that make up the external surface called exo-pinacoderm) functions as a stable sheet of cells with properties for skeletal support and protection by the outer and for transport of nutrients by the inner epithelia, respectively. More specifically, adherent junctions are formed in the epithelia of all sponge groups but their ultrastructure is nowhere the same as the one in eumetazoa. And although all sponge groups show fine septae in their epithelial junctions, distinct septae junctions are only known in calcarea, while in demosponge and homoscleromorph tissues the ladder-like structure of the septate junctions is particularly faint (Leys and Riesgo, 2012). However, it has been shown that demosponge outer epithelia do seal and control the ionic composition of their ECM (Adams et al., 2010). Also, homoscleromorphs epithelia are the only ones that possess a distinct basement membrane (Ereskovsky et al., 2015).

Overall, all sponge epithelia seem to demonstrate a degree of structural integrity and a barrier function in the sense that they are able to control the transepithelial passage of ions (see Leys et al., 2009).

### Development

Porifera have also a simple body plan compared to the eumetazoa, and similarly to the Placozoa, the adults also lack anterior-posterior (AP) polarity (however, the larvae do swim directionally), typical neurons and muscle cells, and a central gut. Sponges possess a rich developmental gene toolkit with many transcription factors, however, their body plan lacks symmetry and it is quite indeterminate (i.e., without fixed number of morphological characteristics in a standard arrangement). Several of their transcription factors are even involved in determining and differentiating muscles and nerves (Srivastava et al., 2010). However, similarly to Placozoa, sponges do not develop a neuromuscular system (Mah and Leys, 2017). Sponges are undergoing an intermediate ‘epithelial development.’ All sponges but the homoscleromorphs lack basal lamina, while no sponge contains the planar-cell-polarity-inducing *Wnt* non-canonical pathway, which is characteristic of diploblasty (Newman, 2016b). Consequently, in Porifera, blastulation, gastrulation, and histogenesis are not quite distinct (Ereskovsky, 2010). In addition, it is still undecided whether sponge cell layers are homologous to eumetazoan germ layers, as they do not undergo progressive fate determination (Degnan et al., 2015). This means that it is highly likely that the existence of a regulatory network guiding progressive germ layer determination (through gastrulation) and the related capacity for diverse cellular differentiation and integration are characteristics of eumetazoan organizations (Nakanishi et al., 2014). Moreover, most sponge cells reside within the mesohyl to which they bind via integrins and within which they can move freely past one another. Such mainly mesenchymal organization (absent in diploblastic Cnidaria and Ctenophora) makes the sponge cells subjects of morphogenetic capacities and dynamics different than those of genuine epithelial tissues (Newman et al., 2009; Newman, 2016a for details).^15^ Therefore, even if some sponges can undergo morphogenetic processes very similar to those met in ‘epithelial development,’ it is surely a development with such a regulatory capacity (by the interplay of epithelial morphogenetic movements and intercellular signaling) that cannot provide standardized body plans. However, contrary to the Placozoa that lack molecules involved in the transduction of the Notch signal (Srivastava et al., 2008), the appearance of the complete *Notch* signaling pathway appears to operate as an intercellular regulator of alternative cellular fates within a common tissue layer (Ehebauer et al., 2006), resulting thus in the high number of cell types exhibited by the Porifera (as opposed to the Placozoa).

## Placozoa

*Trichoplax*^16^ (small, flat marine animals) exhibits a motile feeding mode based on external digestion by a direct endocytosis at the surfaces of ventral epithelial cells (Smith and Reese, 2016). Although placozoa use their cilia to glide over patches of algae that are digested extracellularly, once the nutrients have been ingested the animal starts to change its shape and to make churning movements by contracting its fiber cells in order to help digestion and circulation of the nutrients (Smith et al., 2015). How is this behavior supported organizationally?

### Cell Types

There are six cell types in *Placozoa* with five of them located in the ventral epithelium. The small ventral epithelial cells are the most widespread having apical junctions joining them into an epithelium, but they lack tight junctions and a basal lamina (Smith et al., 2014). Lipophil cells are newly recognized cells that are interspersed regularly within the ventral epithelium and are a good candidate for digestive cells as they are likely to secrete digestive enzymes. Gland cells is another type of newly recognized cells that, contrary to the lipophils, are widespread around the rim of the ventral epithelium but sparse in its central regions. Gland cells seem to be neurosecretory chemosensory cells playing a role in feeding as their secretion modulates the activity of the ventral ciliated and lipophil cells in the presence of food (Smith et al., 2015). Fiber cells are found in a single layer in the interior of *Trichoplax*, and they are also in contact with the dorsal epithelium. These cells are usually considered to be forming a connective tissue through syncytial junctions (a fiber syncytium). This syncytium resembles the excitable epithelial cells in glass sponges though its conduction efficiency and its contractility are uncertain (Ruppert and Barnes, 1994). The upper (dorsal) epitheloid layer contains epithelial cells with junctions as the ventral cells but with fewer microvilli. In most strains of Placozoa each one of these dorsal cells contains a large spherical lipid droplet thus resembling some of the chemical properties of the large lipophil cells. Crystal (birefringent) cells have been lately reported to be found around the entire rim of *Trichoplax* lying underneath the dorsal epithelium near the fiber and lipophil cells and in contact with the fiber cells. It is hypothesized they could be either stem cells or they might have some sensory function as there are reports that Trichoplax could be able to respond to light (Smith et al., 2014).

### Sensorimotor Coordination

In *Placozoa*, the ciliated cells of the ventral epithelium are clearly the effector cells that are responsible for gliding motility. *Trichoplax* will pause much more frequently and for longer time when food is present in the environment. Accordingly, there appears to be a way of sensing the underlying algae as well as triggering of the secretion of granules from the lipophil cells for the algae’s lysis. The gland cells (those interspersed in the inside of the animal together with other ciliated ventral epithelial cells) are supposed to be chemosensory cells that are modulating the activity of the ciliated ventral and of the digestive cells (Smith et al., 2014).

The gliding movements (either for migration or rotation in place) of *Trichoplax adhaerens* are propelled by the asynchronous beating of the cilia of the ventral epithelial cells. The direction of these movements can be changed by the reorientation of the strokes of the animal’s cilia (Smith et al., 2014). The participation of all cells in these movements is not due to any active coordination in between those cells. Probably, each individual ciliated cell senses and responds independently to a chemoattractant, while collective movement toward the food target is ensured by the system of adherens junctions that holds neighboring cells in the two epithelia that enclose the animal and by elastic forces arising from other cells with which they are linked directly and via intervening cells they thus holding the whole animal together during crawling^17^ (Smith and Reese, 2016; Smith et al., 2019). When the animal finds itself over algae the gliding stops as it pauses to feed. Its cilia at the entire ventral epithelium stop beating and specialized cells (lipophils) that are widely separated with each other simultaneously start secreting large ventral granules that lyse the algae. In the absence of a nervous system there must be some other type of spatio-temporal coordination of cellular activities during pausing of locomotion and feeding. The gland cells located at the circumference of the ventral epithelium are chemosensory neurosecretory cells that coordinate the uniform arrest of cilia beating by secreting endomorphin-like peptides upon contact with the algae. Specifically, the peptides are secreted as a gradient in the epitheloid environment and they spread by diffusion. The signals are amplified through a type of positive feedback loop relayed among the secretory cells. It is in this way that ciliary arrest spreads across all over the animal (Senatore et al., 2017).

### Physiology

Similarly to Porifera, Placozoan cells are in constant contact with the water. Placozoa have two layers of cells with the in-between substance being (practically) seawater. In Placozoa the food is ingested through the churning movements of the ventral epithelium that forms a primitive ‘digestive bag.’

The coordination of external digestion through the secretion of granules by the lipophil cells is done through a highly localized secretion that happens by paracrine signaling pathways either based on peptides by the gland cells found in the periphery or by other cells (Smith et al., 2015). Once the group of lipophils have simultaneously secreted their digestive granules, groups of (epithelial ventral and fiber) cells located in the center of the Trichoplax will begin to make elliptical churning movements in order the lysed algae to be endocytosed by the ciliated ventral epithelial cells. The digestive movements resemble those of myoepithelial cells, and their coordination could be realized based on the (so far) suggested contractility of the fiber cells that, to some extent, seem to be interconnected by electrically syncytial conductive junctions (like the ones found in glass sponges) and are connected to all other cell types (Smith et al., 2014). Again, the adherens junctions of the epithelium hold the cells together during digestive movements.

### Development

Placozoa have a very simple body plan compared to the other metazoa. They lack anterior-posterior (AP) polarity, they have no neurons neither muscle cells, and they also lack a proper gut. Contrary to the low morphological complexity of *Trichoplax*, and while its genome is relatively small, the gene content is complex containing genes that specify structures and processes such as ECM production, germline sequestration and neural signaling that are characteristic of higher animals but are absent in *T. adhaerens* (Schierwater et al., 2009). This low morphological complexity in the presence of complex gene content is possibly due to the absence of ‘epithelial development’ and with developmental regulation taking place (mostly) at the cellular level and far less at the whole animal. Specifically, although the cells of the epithelium in Placozoa exhibit apico-basal (AB) polarity, thereby being able to laterally attach to their neighboring cells while also being basally attached to acellular surfaces, they lack a basal lamina—a specialized ECM necessary for a complete epithelium and for the formation of cell sheets in metazoa as it regulates cell behavior and function in tissue genesis and homeostasis (Fidler et al., 2017). This prohibits the epithelia in Placozoa to exhibit interesting morphogenetic movements that will provide germ layers and consequently the capacity for distinct and diverse morphological outcomes in a body plan (Newman, 2016b). Moreover, the epithelium in Placozoa is leaky, lacking any septate and gap junctions (Smith and Reese, 2016), thus not enabling efficient and rich intercellular signaling that could hypothetically mobilize various morphogenetic movements. Consequently, in spite the rich and complex developmental gene toolkit, development in *T. adhaerens* provides a non-standardized and indeterminate body plan (i.e., without fixed number of morphological characteristics in a standard arrangement), with no A/P polarity, and a few cellular types.^18^

## Ctenophora

*Ctenophora* are fed through a combination of swimming and retracting of their two branching tentacles. Swimming is realized by the coordinated beating of the cilia of the comb plates (ctenes). Preys that touch the tentacles that are usually trailing behind the animal (or are extended like a net) are entangled and stuck by the colloblast cells (the major component of the tentacle epidermis)^19^. The tentacle will then retract and the prey will be transferred to the mouth for consumption. In ctenophores with reduced tentacles of reduced size (such as lobate adult animals) or with no external tentacles (such as the Thalassocalycida) food is captured through the oral lobes or through a rapid bell contraction of the peripheral sphincter muscle of its medusa-like body, respectively (Swift, 2009).

Apart from the feeding process described above, ctenophores have an interesting repertoire of motility-based interactions with the environment such as forward swimming, escape swimming, and geotaxis. Let’s describe the complex bodily organization behind such behaviors.

### Sensorimotor Coordination

Ctenophores have an apical sensory organ that is supposed to be the main sensory organ region due to the high concentration of nervous elements surrounding it. It includes a gravity-sensing statolith, mineralized lithocytes that together with balancer cilia serve as mechanoreceptors (sensitive in changes in pressure), and the lamellate bodies that are supposed to be sensitive to changes of light. The main effectors related to the locomotion (swimming) of the animal are the cilia of their comb plates, while true smooth muscle cells^20^ are used for maintenance of the body structure as well as for catching and digesting preys. In all motile interactions the muscles are used to maintain body shape while the animal is moving forward by the beating of cilia as well as for the contraction of the tentacles, the ingestion of food and the egestion of waste in and out of the pharynx, respectively. The coordination of the effectors in all these behaviors is realized through hydrodynamic (beating of the cilia in comb plates) conduction or/and through electrical (in the case of mechanoresponses of the balancing system, of ciliary inhibition for changing of direction of swimming and escaping or for feeding) conduction via the nerve nets. In general, there is a more confined and local (and only indirectly global) and less directly global through-conducting modulation compared to the Cnidaria (Tamm, 2014; Simmons and Martindale, 2015).

### Physiology

Ctenophora, whose body is, similarly to Cnidaria, composed of two epithelial layers (the outer epidermis and the inner gastrodermis) and the jelly like mesoglea in between, have a similar but more elaborated digestive system than Cnidaria. Indeed, the digestive tract of Ctenophora is complete. They have a through-gut with mouth and separate anus. There are two anal pores at the anterior end of the organism for excretion of (mainly) soluble wastes. The pharynx connects the oral with the anal openings via the center of the gastrovascular cavity. Its morphology enables a much more efficient food processing and distribution compared to Cnidaria. The food is processed in three phases as it passes through the acidic environment of the pharynx and then it moves through one alkaline phase along the folds of the pharynx and through another one near the so-called stomach, where food particles are broken down by cilia. The small particles are distributed to the body by the endodermal system of meridional canals that run subjacent to the comb rows, while the remaining larger (mainly exoskeletal) particles will be egested back out of the pharynx (Bumann and Puls, 1997; Tamm, 2014, 2019).

Food digestion and nutrients distribution is realized in a complete digestive tract and it mainly involves cilia beating^21^, and secondarily, epithelial and muscle cells. The esophagus is lined up by cilia pointing in opposite directions on its two sides. The continuous beating of the cilia makes the esophagus a gastric mill that cuts pre-digested prey into small particles, which are then directed to the stomach again through cilia. In some species (beroids) the ingested prey is cut in pieces through the peristaltic waves of muscle contractions. Food particles are distributed via the branched gastrovascular canal during brakes between bouts of defecation. Indigestible fragments are egested through the mouth and small waste particles are egested through one of the two anal pores (defecation). Ingestion and egestion cannot happen simultaneously as bouts of defecation are shutting down food handling and distribution in the digestive system. Localized tracts of opposite beating cilia line up all canals. Defecation is preceded by the combination of the constriction of the walls of esophagus together with the slowing down of cilia beating. Although these systems haven’t been thoroughly studied, there is experimental evidence that the adjustment of the ciliary beating in opposing directions and the various muscle contractions for food ingestion/digestion and fluid circulation, as well as the modulation of cilia beating prior to defecation is likely to be regulated by local electrical conduction realized through epithelial gap junctions or by neural nets (see Tamm, 2014, 2019 for details; Simmons and Martindale, 2015 for a relevant review).

There is an apparent absence of classic neurotransmitters (i.e., acetylcholine, serotonin, histamine, dopamine, adrenalin, noradrenalin, and octopamine) and of neurotransmitter receptors in Ctenophores compared to the Cnidaria (Moroz et al., 2014). However, there is glutamate and GABA, acetylcholinesterase is present in *Mnemiopsis leidyi* (Ryan et al., 2013), while acetylcholine (together with adrenaline) have been shown to play a role in *M. leidyi* bioluminescence (Marlow and Arendt, 2014). Ctenophores also contain many G-protein-coupled receptors and several neuropeptide precursors (Moroz et al., 2014). Moreover, ctenophores express a diversity of innexins (also used to form gap junctions in Cnidaria), and opsins necessary for a sensory neuron for photo-reception (Jekely et al., 2015). All these findings suggest the existence of a complex peptidergic armory in the Ctenophores nervous system that, similarly (though likely not that widely and globally) to the Cnidaria regulate the animal’s physiology.

### Development

Ctenophores have several unique morphological features that distinguish them from all other animals. They are diploblastic epithelial animals with a gelatinous mesoglea penetrated by true muscle cells, nerves, and mesenchymal cells. They have a branching ciliated gastrovascular system that runs the body along the oral/aboral axis. They consist of four quadrants separated by the tentacular and the esophageal plane. There are two comb rows, a half of a tentacle and a quarter of the apical organ in each one of the quadrants. Since adjacent quadrants are not morphologically identical to each other (anal canals are found only in two diagonally opposed quadrants) ctenophores have a rotational symmetry (this is neither a radial nor a bilateral one) (Martindale and Henry, 2015). Ctenophora don’t have as rich developmental genetic toolkit as Cnidaria. Indeed, the gene content of ctenophores is much more similar to the one of sponges than it is to any other metazoan (Martindale and Henry, 2015).^22^ Ctenophora—most likely because they are holopelagic and thus they should get to a free-living feeding stage very quickly—are (contrary to Cnidaria) direct developers. The polarity of the primary axis is provided by cytoplasmic maternal products, while the cWnt signaling pathway appears to be important in the establishment of polarity later in their development of ctenophores (Pang et al., 2010; Jager et al., 2013). Also, although both their epithelia form basal laminas—contrary to Cnidaria, where the basement membrane plays numerous important roles in directing cell differentiation, cell migration and tissue regeneration through intercellular signaling—the basal lamina of Ctenophores seem to play only a structural role rather than a role in cell signaling (Babonis and Martindale, 2016). Therefore, although Ctenophora exhibit epithelial development and a standardized and determinate body plan as Cnidaria do, it seems that their development is regulated differently than in Cnidaria, demonstrating a more egg-based stereotyped regulation than a developmentally rich regulation that endogenously emerges as development unfolds.

## Cnidaria

The rest of the animals (belonging to the Eumetazoa) are macrophagous, i.e., they can be fed on large items through a gut. In this case food is inserted inside the body but it is digested outside of the cells. It is in macrophagy that motility becomes extremely important because for a gut to work the body must be positioned in relation to food items (locating, catching and directing them in the mouth/gut), which involves a sensorimotor process that actively and precisely manipulates the environment at the bodily scale.

In general, Cnidaria have the capacity for diverse motility-based interactions with the environment that are mainly manifested as highly directional movements of various parts of their body that in several species (jellyfish) is combined with swimming with increased maneuverability. Cnidaria catch their preys through a combination of the internally generated fast rhythmic movements used for swimming with the sharp and directed movements of their tentacles^23^ and the timely activation of nematocysts—which are discharged upon a prey touches the tentacles, thereby capturing or paralyzing it.^24^ The swimming is realized through the contraction of their bells in several different rhythms, thus reducing the space inside the rim of the bell, while forcing water out through the opening, and the springiness of the mesoglea (the jelly like part of the Cnidarian body) powers the recovery stroke. What is the bodily organization behind all this interesting behavior?

### Cell Types

Cnidaria (as well as ctenophores) have almost a similar number of cell types as porifera (Bell and Mooers, 1997; Carroll, 2001), however, their cellular diversification is different, and this is strongly related to the difference in the mode of feeding. Passing from distributed microphagy to a more centralized external digestion without a gut to an external digestion within a gut cavity (macrophagy) there is a need for distinct cells that circulate nutrients (through contractility), uptake, and excrete nutrients in the internal cells of a three-dimensional body. Apart from the feeding-related cells in Cnidaria and Ctenophora we also find distinct cells related to the sensorimotor behavior of the animal such as myoepithelial cells or pure muscle cells (in the case of Ctenophora), nerve cells (mostly arranged in nerve nets) and several sensory cells (see text below for cnidaria and in section 5 for Ctenophora, but also Arendt et al., 2015).

### Sensorimotor Coordination

Jellyfish have primitive ‘sense organs’ that form part of the epidermis. They consist of neurosecretory cells and of small arrays of sensory neurons, which function as sensory and motor-neurons that establish bi-directional synapses (mainly) with myoepithelial cells and nematocysts, thus providing particular sensory modalities—i.e., light reception through simple eyes, chemoreception through epithelial sensory cells, sensation of gravity through statocysts, and mechanoreception (touch and vibration) through cilia and cnidocytes or nematocytes (see Jacobs et al., 2007 for details).

The activity of all moving parts (bell and tentacles) is realized through the cnidarian musculature that is formed by myoepithelia^25^ that together with the epithelial tissue are the effectors that provide coordinated (synchronized) local contractions. However, this comes with important limitations on the variety of patterning possibilities (i.e., the degree of plasticity and flexibility achieved by excitable myoepithelia)^26^. The cnidarian body overcomes this limitation with the differentiation of a nervous system (NS), which is composed of distributed nerve nets associated with simple sensory receptors that are distributed radially around the body of the animal.^27^ The effect of this arrangement of nerve nets permits the generation of different action potentials by the rapid communication of a stimulus (through the release of neurotransmitters) from a part of the animal to any other part, over relatively long distances and with a significant degree of modulation, which is not possible in animals lacking neurons. This allowed for both quicker responses and the enhancement of functional diversification of the myoepithelial patterning capabilities.

As described above, Cnidaria have primitive ‘sense organs’ that form part of the epidermis, and which consist of neurosecretory cells and of small arrays of and sensory neurons operating in certain different modalities. However, none of these sensorial modalities could be put into work without the existence of a nerve net operating as an intermediate layer integrating the sensory stimuli with several motor outputs in a timely and diverse manner. Nerve nets are used to support and integrate multiple distinctly different actions generated by different patterns of neural activity (Satterlie, 2008). In the hydrozoan jellyfish *A. digitale*, for instance, some parts of the nerve net are fused forming longitudinal or circular tracts innervating its swimming muscles thus allowing very fast signal conduction, which in turn can support fast attack or escape reactions or slow rhythmic swimming for feeding. The centralization of the nervous circuitry in *A. digitale* and in other hydrozoans has resulted in the formation of two nerve rings along the bell margin (inner and outer), both of which play the role of extra distinct neural components in the modulation of its motility. The outer nerve ring is connected to the sense organs and has a sensory function, whereas the inner nerve ring has a motor-sensory function, regulating the contractions of the umbrella as a pacemaker (Satterlie, 2008). This instance of implicit functional subdivision in *A. digitale* results in an underlying neural circuitry, in which the fast escape part can override the part responsible for the regular swimming but the former part can also be co-opted by the latter for its own uses (Satterlie, 2002, 2008).

### Physiology

Cnidaria (and Ctenophora) have two epithelial layers of metabolically active cells separated by the mesoglea (a metabolically inert substance) thus achieving diffusional contact with seawater for each one of their cells. Contrary to sponge and placozoa, in cnidaria (as well as in ctenophora) there are primitive organs related to the transportation and absorption of nutrients. In cnidaria the endoderm (or gastrodermis – one of the two epithelial layers) forms an internal body cavity called the gastrovascular cavity that lines the gut forming thus a proto-coelenteron. Intake and excretion of materials in and out this gastrovascular cavity happens through a single opening that serves as both mouth and anus. This allows for the consumption of greater food size compared to placozoa and porifera. The coelenteron is almost completely closed and separate from the environment and digestive enzymes can be released and concentrated within this cavity. Food is thus extracellularly digested within the gastrovascular cavity and then it is intracellularly digested within each gastrodermal cell. The transport of digested (soluble) nutrients happens intercellularly via the epithelial cells.

The NS in Cnidaria (together with other epithelial cells) regulates various parts and aspects of their physiology via a neuroendocrine-like activity (Tarrant, 2005). Cnidaria achieve physiological regulation thanks to neuroendocrine^28^ cells synthesizing peptide-signaling molecules (acting as neurotransmitters or/and neurohormones)^29^ whose circulation occurs primarily through epithelial diffusion, and which regulate a variety of physiological processes such as ingestion of prey, digestion, and excretion of nutrients, oocyte maturation, the pumping activity of the body column, and spawning.

### Development

Cnidaria have a more complex body plan compared to the Placozoa and to the Porifera. They are diploblastic, radially^30^ symmetrical animals with an oral/aboral axis, a subumbrellar cavity (that is not a coelom), and primitive organs (gut, pharynx, tentacles, and sense organs), and of course, an epithelio-neuromuscular structure. Notwithstanding the richness of the developmental genetic toolkit of placozoa and especially of sponges, the developmental toolkit of Cnidaria is richer than the one of all other basal metazoa in all important aspects characterizing the metazoan body plans such as morphogenesis, regional tissue patterning, axis formation, and cell-type specification.^31^

But equally importantly and complementary to the rich developmental gene toolkit is the fact that Cnidaria possess two layers of complete epithelia that enable their embryonic multicellular masses to clearly exhibit gastrulation and PCP-based body elongation (see Newman, 2016b for details and references therein) in a fashion that in turn allows an efficient and rich intercellular signaling, which propels the further mobilization of various morphogenetic movements providing thus a standardized and determinate body plan with a fixed number of morphological characteristics in a standard arrangement.

The role of the NS is also of great importance in a variety of developmental processes. Specifically, neuropeptides are regulating a variety of developmental and physiological processes, such as the induction or inhibition of neural differentiation, neurogenesis, and oocyte maturation, and morphogenesis (Tarrant, 2005; Takahashi and Takeda, 2015).

## Discussion: A Thesis of Bodily Complexity

Typical ‘complex adaptive bodies’ (CABs) with their immense behavioral potential are a very successful evolutionary invention. However, the preceding evolution of the basic configuration of such typical animal bodies involved a set of evolutionary changes that could not have been driven by the eventual fitness options that, after all, only became available when these basic ingredients were in place. In Section “Setting the Stage for Bodily Complexity: Contraction-Based Motility Requires Integrated Bodies and Vice-Versa,” we presented and we argued why the animal sensorimotor organization (ASMO) is such an evolutionary key transition. This organization is so familiar to us that it becomes almost invisible and hard to appreciate as a separate achievement of life. Still, it amounts to a major and specific evolutionary transition. The road traveled to this evolutionary point (ASMO) is not straightforward but dependent on bringing the central ingredients together in a specific form of organization.

What is characteristic of this evolutionary road is the transition from cilia-based to contraction/muscle-based motility. The latter enabled large and fast organisms that could move about as large motile agents, which resulted in animals being the dominant macroscopic life-form together with plants. So, organizing bodies capable of motility mediated by muscle-based (and myoepithelial) contractility is a key feature that must have been in place for large and fast animals to become possible. But muscle-based motility itself depends on the combination of many other factors being integrated into a multicellular organization as a single unit. More specifically, as we show, the transition from widely available (and used) cilia to muscle-based motility is a major one that involves: the presence of a body that relies on contraction for reversible motility; developmental processes that generate complex and standardized body forms; a nervous system that coordinates the whole organism in a way that is fast and unifying; complex physiology to keep all cells involved alive and thriving by keeping the whole body ‘energized’ and oxygenated and that weighs these needs depending on the requirements of the moment (active or resting, etc.); a molecular interface (a gut or what is present for this purpose).

However, as we show (in Sections Porifera, Placozoa, Ctenophora, and Cnidaria) in our discussion of the bodily organization of the four known basal animal phyla and the eventual space of possible multicellular configurations toward ASMO, contraction-based motility is not a self-evident switch from cilia but something that is just one of the many options available for the use of tissue contraction that was present, and which in all these cases is used in some way to change their body configuration and to control their feeding, though in very different ways. Porifera are mainly sessile, and use contraction to control their water canal system, not to move about or manipulate their environment. Placozoa move around by using cilia while exhibiting contractions to change their body shape, and presumably to help digest their food. Neither have standardized bodies in contrast to both Cnidaria and Ctenophora.

Ctenophora have a standardized diploblastic epithelial body with muscles and a NS. However, they appear to have a major organizational difference with respect to how they use their contractions compared to the Cnidaria; Ctenophores rely on cilia as a prime mover of the body, while contraction is mostly used to maintain body shape and (only) secondarily to coordinate the through gut. This is something very different from cnidarians (and bilaterians), which are fully depending on muscle control to interact with their environment. In addition, Ctenophora appear to exhibit less intercellularly regulated development than the Cnidaria since epithelial intercellular signaling doesn’t seem to be very operative during their (at least) early developmental stages and their genetic developmental toolkit is significantly decreased (compared to the Cnidaria) from the typical bilaterian genome (Martindale and Henry, 2015, see also footnote 22). Also, the genomic content of nervous system genes is the most reduced of any animal (Simmons and Martindale, 2015).

From a merely biological point of view, these aspects do not necessarily have positive or negative implications. After all, nature has been always exploring several different multicellular organizational forms. However, from the perspective of the evolution of biological organizations (especially when one considers the road toward ASMO), there is a notable implication of the different integration exhibited by the Ctenophora (compared to the Cnidaria). In particular, when one considers the fact that Ctenophora have the most advanced (and complete, though transient) digestive system of all four basal phyla, this suggests that their bodily organization uses tissue contractility much more for the shaping of the animal and the manipulation of digestion than it does for interaction. As a matter of fact, as Tamm (2014) notes, in contrast to the global functionality of nerve nets and myoepithelial conduction that can trigger behavioral responses anywhere on the animal and spread them to all related effectors, the neuromuscular organization of ctenophores is spatially confined and interacts only indirectly with other biomechanical and neural conducting pathways. Consequently, as ctenophores move by cilia, which is characteristic of unicellular organisms, and exhibit a rather minimal presence of reversible, contraction-based changes in body-shape, leave alone in body appendages, we conclude that they exhibit an ASMO only in a basic and atypical way.

So, while contractile free-movement is key and the capacities for contraction are present from the epithelial start, their use for motility is hard. And while the use of cilia can be seen as generating a local optimum, there is, as we show, no straightforward pathway from the presence of contractile epithelia to contraction-based motility. This would require an evolutionary benefit over cilia that are much easier to use and control. But getting to this evolutionary point, is difficult because, as we argue, it first requires the fulfillment of various organizational conditions.^32^ This is exactly what happens in the Cnidarian organization.

In Cnidaria, the whole body is hold together due to its genuinely epithelial nature. Nervous systems operate as a regulatory sub-system that spatiotemporally coordinate the combinatorial execution of local contractions of distant groups of epitheliomuscular cells throughout the whole animal body, thereby maintaining sensorimotor loops. Cnidaria show a diverse repertoire of movements in which either the whole body (in jellyfish), its parts or several combinations of them (in all species) can perform fast, reversible, and well-coordinated movements. This coordination results in a functionally rich behavior only because it is integrated in a body plan with a set of primitive and differentiated organs that provide the animal with the proper physiology (metabolic and biomechanical requirements) for its behavior. As discussed in detail (see Section Cnidaria), the common basis of integration between sensorimotor and physiological coordination is the diploblastic epithelial organization and the NS that, together with an abundance of neuro-transmitters, neurohormones and non-neuronal hormones, modulate physiological processes and ensure global homeostasis and growth. The emergence of such a coordinated and standardized body is only possible through specific developmental regulation exhibited only in a genuinely epithelial development (Arnellos and Moreno, 2016), where the combination of epithelial morphogenetic movements with the rich intercellular signaling provide a continuously unfolding system coordinating the development of such complex bodies (Arnellos et al., 2014). Again, the NS (together with various neuropeptides) is actively modulating the development of several (even late) developmental stages of the adult Cnidaria. This is exactly how the neuro-epitheliomuscular Cnidarian body achieves such as strong integration between its behavioral, physiological and developmental processes.

The bodily complexity (and the related form of integration) exhibited by Cnidarian organizations has several implications. A most prominent one is that Cnidaria show motility-based interactions that are completely new (and thus different) compared to the ones demonstrated by their unicellular parts. This is not the case neither for the Porifera, where most of their contraction-based actions are similar to the ones of choanoflagellates nor for the Placozoa where motility is done by cilia and digestion is external just as the case of unicellular eukaryotes, nor quite exactly for the Ctenophores which, after all, they still move via cilia. In this way, the Cnidarian organization makes *a qualitative jump—a major transition—*regarding the capacity of a multicellular body for contraction-based interactions compared to the other early metazoa. In the account we sketched, the Cnidarian body exhibits the special organizational conditions necessary for muscle-based contractions to be reused as a motile force. This is why Cnidaria show an unequivocal ASMO. Consequently, of the four phyla discussed, the cnidarians come closest to the features that are central to the bilaterian ‘complex adaptive bodies’ (Trestman, 2013). They exhibit a specific form of integrated organization, where multicellular contraction and its sensory potential are fully established, even when the various senses remain rather limited, barring a few exceptions.

The events constituting the evolutionary transition that resulted in modern animals, including the bilaterians, happened a very long time ago and we do not yet have a sufficiently clear grasp of these events (e.g., Erwin, 2015). In order to assess some of the possibilities for basic animal configurations, we looked at the characteristics of the few early diverging phyla that still have living representatives. As we are all highly familiar with typical bilaterian animals, it may seem that this configuration is actually the most natural and normal one. By surveying the four basal animal phyla, we hope to have shown that this is not the case and that the basic features that are, to some extent, present in all animal phyla, can come together as functioning organisms in very different ways. In this way an organizational account helps to set aside bilaterian-influenced descriptions of non-bilaterian animals. Moreover, it seems that the evolutionary road toward a muscle-based system of motility is not a straightforward one. The ability to use contractions for efficient movement and sensing at a multicellular scale—which must be considered the key to the major success of bilaterians as typical animals—can only be achieved on the condition of a complex, highly differentiated and specifically integrated bodily organization. Getting such a complex bodily organization in place sets up a major evolutionary bottleneck when it comes to the evolution of typical animals.

## Author Contributions

AA and FK contributed equally to the conception, design, preparation, and writing of the manuscript.

## Conflict of Interest

The authors declare that the research was conducted in the absence of any commercial or financial relationships that could be construed as a potential conflict of interest.

## Footnotes

1 Here we discuss what is *typical* for animals and set aside various exceptions. With ‘typical’ we refer to free-moving animals like vertebrates, arthropods, jellyfish, etc. These typical features are not present in all animals, in particular the most basic animal phyla. Nor do we deny that many animals are sessile, live in colonies, such as coral reefs attest, or are part of multi-multicellular entities such as the Portuguese man o’ war.

2 For the theoretical background of ASMO see Keijzer (2015).

3 Our intention is not to discuss each cell type analytically (after all, there is no such complete record) but to mention the cell types required for the realization of body motility and the related physiology in the transition from microphagy to macrophagy. Several cell types will be mentioned in Sections “Porifera,” “Placozoa,” “Ctenophora,” and “Cnidaria.”

4 Epithelia are generally considered as cells clonally derived from a fertilized egg that remain attached to one another by cadherin-based membrane proteins, and which are at the same time being independent while also being attached to each other thus forming embryonic tissues that behave like liquids (Newman, 2016b).

5 For the moment, we are just stating the common aspects of what we refer to as ‘epithelial development’ that characterizes all metazoa. However, as we have previously explained, a genuinely epithelial developmental organization is to be met only in the Eumetazoa (Arnellos and Moreno, 2016). As we explain in Sections “Porifera,” “Placozoa,” “Ctenophora,” and “Cnidaria,” the developmental process is coordinated in different ways in the four early metazoa, resulting thus in body plans with crucial differences in their complexities.

6 Again, striking absences in the genomes of each of the four early metazoa lineage and their implications in development of the body plans will be discussed in Sections “Porifera,’ “Placozoa,” “Ctenophora,” and “Cnidaria.”

7 Food particles are taken up by phagocytosis or pinocytosis followed by intracellular lysosomal degradation (Leys and Eerkes-Medrano, 2006).

8 The pinacoderm functions as a stable sheet of cells with properties for skeletal support and protection by the outer and for transport of nutrients by the inner epithelia, respectively.

9 The choanocytes form a round chamber and beat their flagella to draw water from the incurrent endopinacoderm-lined canals into the chamber where bacterial food are filtered by the microvilli. The water is then expelled through the osculum (see Ruppert and Barnes, 1994 for details).

10 Glass sponges (Hexactinellida) are different from all other sponges because they have a syncytial organization. They cannot contract their “epithelia” because their choanocytes are actually a syncytium.

11 The pinacoderm does not qualify as a muscle tissue. However, it contains contractile filaments traversed by extended actin networks. The elementary contractility of these filaments has been experimentally verified (Nickel et al., 2011).

12 In general, sponge larvae move by independent ciliary beating. However, they rarely perform horizontal displacement by ciliary beating during their dispersal in open waters. Instead, the cilia are only used to keep larvae rotating along their longitudinal axis, which apparently allows for the re-adjusting of the depth range while drifting (see Maldonado, 2006 for details).

13 Propagation along the whole sponge can take from 30 min to a full hour. In general, signaling in cellular sponges is three orders of magnitude slower than neuronal signaling, while the fastest rate of contraction in sponges is still 10 times slower than action potential in plants (Leys, 2015).

14 Choanocytes have a single flagellum surrounded by a ring of microvilli and constitute the choanoderm (the feeding epithelium).

15 The continuous movement of individual cells allows the actively remodeling of the branched skeletal structures in Porifera, thereby yielding a non–precisely determined anatomy in a body form that is shaped asymmetrically and plastically by its environment (Meroz-Fine et al., 2005).

16 This is the originally discovered and most widely described placozoan species. *Hoilungia hongkongenesis* (the second placozoan species) and all yet undescribed species are morphologically indistinguishable. *Polyplacotoma mediterranea* is a newly discovered species that can adopt ramified body shapes. No structural differences to other placozoans have been detected for *P. mediterranea* so far (Osigus et al., 2019).

17 This is an epithelium that lacks any tight or gap junctions.

18 However, little is known about the development of *T. adhaerens* and of Placozoa in general, yet.

19 All ctenophores except the beroids have tentacles and thus possess colloblasts. Another exception is *Euchlora* that does not possess colloblasts but instead has nematocysts that it obtains from the medusae it consumes (Pang and Martindale, 2008).

20 Contrary to the Cnidaria there is no epitheliomuscular component (Hernandez-Nicaise, 1991).

21 In a recent work Tamm (2019) reports that the widening and narrowing of the endodermal canals that play a major role in food distribution and waste elimination are more possibly due to fluctuations in hydrostatic pressure within the canal lumens caused by fluid flow driven by the cilia that are lining the canal walls.

22 Ctenophora lack most of the secreted antagonists to the TGF-B pathway, appear to lack Hox and ParaHox genes (Moroz et al., 2014), the orthologs of several HedgeHog pathway components, and the JAK/STAT pathway. Nuclear receptors (NR) are expressed only after the onset of gastrulation, and the same goes (most importantly) for the canonical WNT pathway, while they have fewer ligands and receptors for the canonical WNT pathway than do cnidarians (Mullikin et al., 2010; Pang et al., 2010).

23 In the case of Anthozoa there is no swimming but predation happens through the use of moving tentacles and their nematocysts.

24 A nematocyst is a stinging organelle contained in the specialized stinging cells (cnidocytes) that are located primarily at the tips of the tentacles, and secondarily in the two epithelial layers (the outer layer called epidermis, and the inner one called gastrodermis).

25 All muscular structures described so far in Cnidaria are epitheliomuscular. Epithelial smooth muscles are generally regarded as primitive features and typical for Cnidaria. Anthozoa (corals and anemones) possess only smooth muscles and striated muscles have only be reported in one very derived clade of swimming anemones. All three classes of Medusozoa are described as possessing both striated and smooth contractile fibers. See Seipel and Schmid (2005) and Burton (2008) for details.

26 The main problem of epithelial conduction in coordinating and synchronizing muscle sheets of effector cells is the lack of directional and selectively targeted propagation of impulses (i.e., an epithelial conduction cannot circumvent an intermediate tissue without activating it, neither can modulate nor regenerate a signal, see Anderson, 1980 for details).

27 Actually, as argued by Mackie (2004), the superficial name of ‘nerve nets’ undermines the neuronal organization of Cnidaria, since some of these nerve nets are considered to reflect a considerable degree of centralization (Satterlie, 2011 for details).

28 The role of neuroendocrine cells in Cnidaria is played by both sensory cells integrated into the epidermis and by sub-epidermal ganglion cells, where endocrine cells are epidermal epithelial cells and gastrodermal neurons.

29 Almost all neurotransmitters, neurohormones and non-neuronal hormones are present in Cnidaria. Most signaling molecules in Cnidaria are peptides, but there are also small non-peptide regulators (Kass-Simon and Pierobon, 2007).

30 Some adult anthozoa even present bilateral symmetry and thus axes differentiation (Leclère and Rentzsch, 2014).

31 Cnidaria have a complete TGF-b signaling pathway, a complete canonical WNT signaling as well as a surprising diversity of WNT ligands, nuclear receptors (NRs), the Notch/Delta signaling cascade, a complete set of HedgeHog signaling components, Hox and ParaHox genes, the RTK/FGF signaling, and components of JAK/STAT signaling (Babonis and Martindale, 2016).

32 This is a characteristic and generic requirement for all biological organizations, namely, their differentiated functionality should first be in place in order for natural selection to be able to operate on them (see Arnellos and Moreno, 2012; Moreno and Mossio, 2015 for extensive discussions).
